# Supplementary material for: Alterations of Lipid Profile in Livers with Impaired Lipophagy
Source: Int J Mol Sci. 2022 Oct 6;23(19):11863. doi: 10.3390/ijms231911863 (PMC9569596; doi:10.3390/ijms231911863)
Supplement: Supplementary file 1 [file ijms-23-11863-s001.zip › ijms-1921868-supplementary.pdf]

Alterations of lipid profile in livers with impaired lipophagy

Wenke Jonas[1,2], Kristin Schwerbel[1,2], Lisa Zellner[1,2], Markus Jähnert[1,2], Pascal Gottmann[1,2], and Annette Schürmann[1,2,3]

[1]Department of Experimental Diabetology, German Institute of Human Nutrition Potsdam-Rehbruecke, 14558 Nuthetal, Germany;

[2]German Center for Diabetes Research, 85764 München-Neuherberg, Germany;

[3]University of Potsdam, Institute of Nutritional Sciences, 14558 Nuthetal, Germany

Supplementary results

Table S1: Fatty acid composition of total lipids in liver tissue of mice with reduced lipophagy (ΔIRG) compared to control mice (IRG). Data are shown as means ± SD and analyzed by unpaired *t*-test with Welch´s correction. Significant values ($p < 0.05$) are bold. IRG, $n = 7$; ΔIRG, n = 7

**Fatty acid**	**(nmol/mg)**	
	**IRG**	**ΔIRG**
C12:0	0.171 ± 0.068	0.217 ± 0.106
C14:0	0.900 ± 0.295	**1.486 ± 0.483**
C14:1n-5	0.127 ± 0.045	0.213 ± 0.154
C15:0	0.216 ± 0.066	**0.378 ± 0.111**
C16:0	43.716 ± 4.897	**63.209 ± 9.039**
C16:1n-7	3.419 ± 0.865	**6.670 ± 1.883**
C17:0	0.345 ± 0.091	**0.644 ± 0.116**
C18:0	15.574 ± 1.585	**18.435 ± 1.934**
C18:1n-9	33.127 ± 7.355	**64.327 ± 11.816**
C18:2n-6	31.466 ± 4.725	**58.269 ± 11.517**
C18:3n-3	2.593 ± 0.437	**5.393 ± 1.311**
C18:4n-3	0.001 ± 0.000	0.001 ± 0.000
C20:0	0.713 ± 0.091	**1.882 ± 0.366**
C20:1n-9	0.724 ± 0.234	**2.663 ± 0.643**
C20:2	0.555 ± 0.125	**1.606 ± 0.314**
C20:3n-6	1.669 ± 0.359	**3.220 ± 0.678**
C20:4n-6	14.769 ± 1.420	15.093 ± 2.240
C20:5n-3	0.658 ± 0.063	**0.892 ± 0.224**
C22:0	0.917 ± 0.139	0.848 ± 0.116
C22:1n-9	0.156 ± 0.016	**0.217 ± 0.031**
C22:2n-6	0.002 ± 0.001	0.003 ± 0.001
C22:4n-6	0.556 ± 0.044	**0.900 ± 0.208**
C22:5n-3	1.481 ± 0.120	**2.173 ± 0.618**
C22:6n-3	15.543 ± 1.562	16.021 ± 2.228
C24:0	0.247 ± 0.054	0.216 ± 0.041
C24:1n-9	0.255 ± 0.041	0.217 ± 0.045
C26:0	0.003 ± 0.000	0.003 ± 0.000

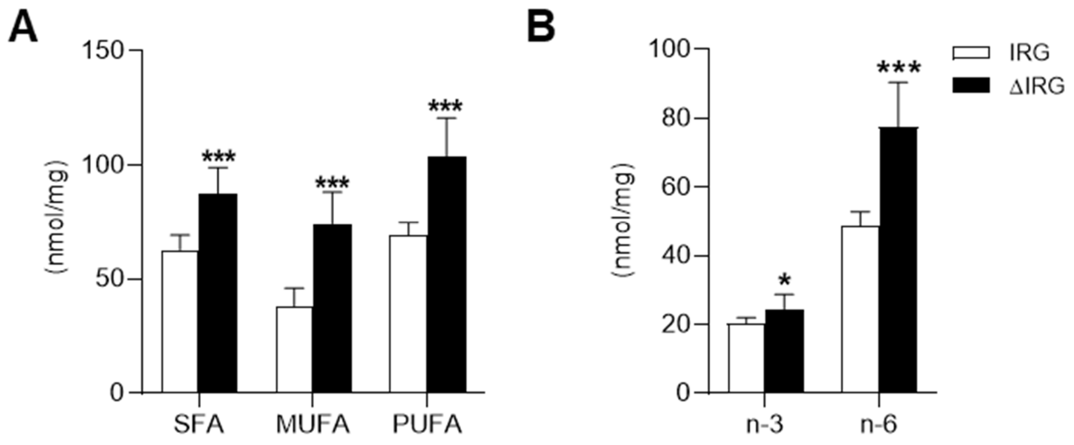

Figure S1. Hepatic lipid content of in total lipids of IRG and ΔIRG mice. (**A**) Concentration of saturated (SFA), monounsaturated (MUFA) and polyunsaturated (PUFA) fatty acids levels. (**B**) Concentration of total fatty acids in n-3 and n-6 series. Data are shown as means ± SD and analyzed by unpaired *t*-test with Welch´s correction. * $p < 0.05$; *** $p < 0.001$; IRG, $n = 7$; ΔIRG, $n = 7$

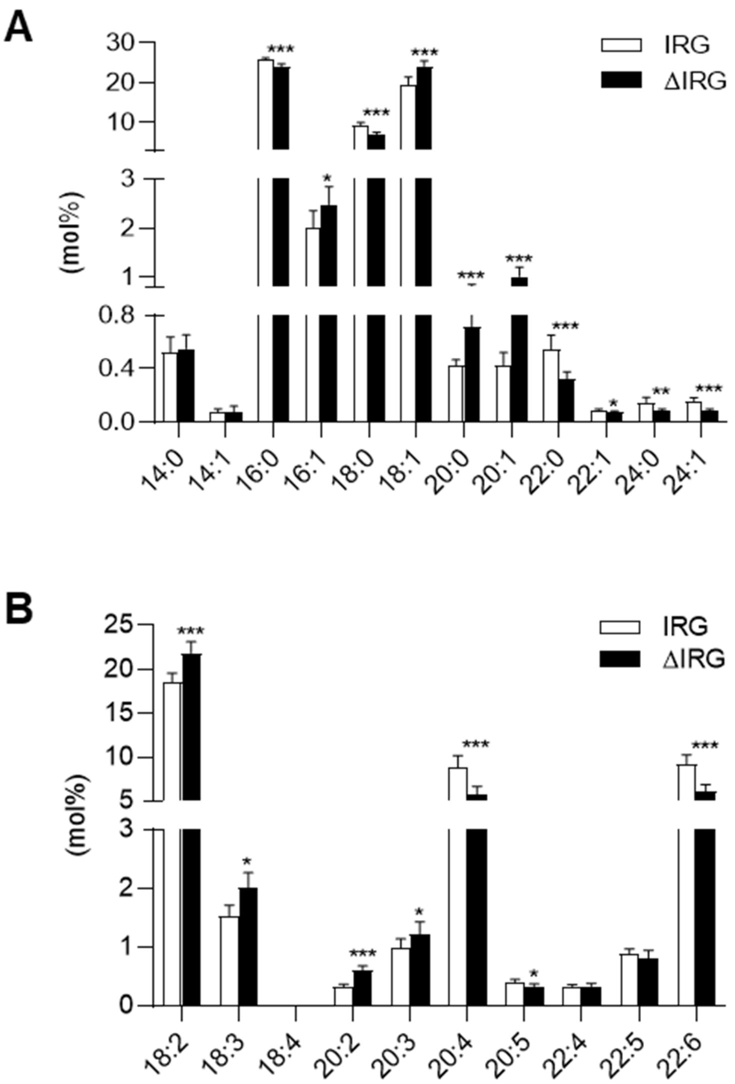

Figure S2. Relative abundance of individual lipid species in total lipids. (**A**) Saturated and monounsaturated fatty acids as well as (**B**) polyunsaturated fatty acids in liver samples of ΔIRG mice compared to IRG mice. Data are shown as means ± SD and analyzed by unpaired *t*-test. * $p < 0.05$; ** $p < 0.01$; *** $p < 0.001$; IRG, $n = 7$; ΔIRG, $n = 7$

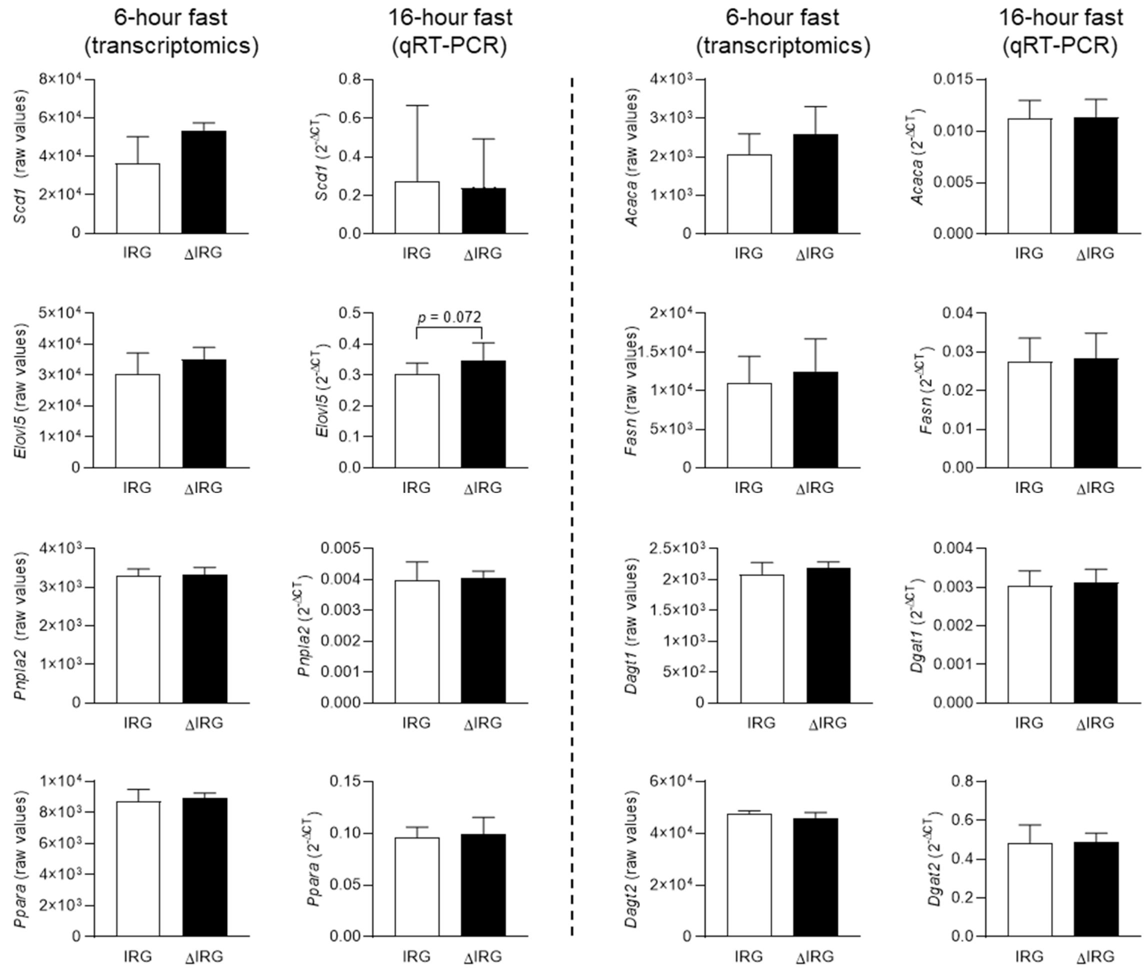

Figure S3. Hepatic expression of genes related to lipid metabolism. Mice were sacrificed after a 6- or 16-hours period of fasting. Gene expression was analyzed by transcriptomics or qRT-PCR, respectively. Data are shown as means ± SD and analyzed by unpaired *t*-test with Welch´s correction. 6-hours: IRG, n = 4; ΔIRG, n = 4; 16-hours: IRG, n = 8; ΔIRG, n = 7.

Supplemental material

Table S2: List of assays used for qRT-PCR

Gene	Description	Assay-ID	Company
Acaca	Acetyl-CoA carboxylase alpha	Mm.PT.58.12492865	Integrated DNA Technologies (IDT)
Dgat1	Diacylglycerol O-acyltransferase 1	Mm00515643_m1	Thermo Fisher
Dgat2	Diacylglycerol O-acyltransferase 2	Mm00499530_m1	Thermo Fisher
Eef2	Eukaryotic translation elongation factor	*Self designed**	IDT
Elovl5	ELOVL fatty acid elongase 5	Mm.PT.58.43010575	IDT
Fasn	Fatty acid synthase	Mm.PT.58.31183175	IDT
Hprt	Hypoxanthine guanine phosphoribosyl transferase	Mm.PT.39a.22214828	IDT
Ifgga2	Immunity-related GTPase	Mm01621208_s1	Thermo Fisher
Ppia	Peptidylpropyl isomerase A	Mm.PT.39a.2.gs	IDT
Pnpla2	Patatin like phospholipase domain containing 2	Mm00503040_m1	Thermo Fisher
Ppara	Peroxisome proliferator activated receptor alpha	Mm00440939_m1	Thermo Fisher
Scd1	Stearoyl-Coenzyme A desaturase 1	Mm.PT:58.43504385	IDT

* Probe: 5´-TAAGCAGAGZENCAAGGATGGCT-3´
Primer1: 5´-TGAGGTTGATGAGGAAGCCC-3´
Primer2: 5´-CACAATCAAATCCACCGCCA-3´
